# Predictors of in-hospital mortality in elderly patients with bacteraemia admitted to an Internal Medicine ward

**DOI:** 10.1186/1755-7682-4-33

**Published:** 2011-10-04

**Authors:** Marta Rebelo, Branca Pereira, Jandira Lima, Joana Decq-Mota, José D Vieira, José N Costa

**Affiliations:** 1 Department of Internal Medicine, University Hospital of Coimbra, Coimbra, Portugal; 2 Department of Infectious Diseases, University Hospital of Coimbra, Coimbra, Portugal; 3 Head of Department of Internal Medicine, University Hospital of Coimbra and Professor in the Faculty of Medicine of the University of Coimbra, Coimbra, Portugal

**Keywords:** elderly, bacteremia, bloodstream infection, hospital mortality

## Abstract

**Background:**

Infectious diseases are a common cause of increased morbidity and mortality in elderly patients. Bacteraemia in the elderly is a difficult diagnosis and a therapeutic challenge due to age-related vicissitudes and to their comorbidities. The main purpose of the study was to assess independent risk factors for in-hospital mortality among the elderly with bacteraemia admitted to an Internal Medicine Ward.

**Methods:**

Overall, a cohort of 135 patients, 65 years of age and older, with bacteraemia were retrospectively studied. Data related to demographic information, comorbidities, clinical parameters on admission, source and type of infection, microorganism isolated in the blood culture, laboratory data and empirical antibiotic treatment was recorded from each patient. Multivariate logistic regression was performed to identify independent predictors of all-cause in-hospital mortality.

**Results:**

Of these 135 patients, 45.9% were women. The most common infections in this group of patients were urinary tract infections (46.7%). The main microorganisms isolated in the blood cultures were *Escherichia coli *(14.9%), Methicillin-resistant *Staphylococcus aureus *(MRSA) (12.0%), non-MRSA (11.4%), *Klebsiella pneumoniae *(9.1%) and *Enterococcus faecalis *(8.0%). The in-hospital mortality was 22.2%. Independent prognostic factors associated with in-hospital mortality were age ≥ 85 years, chronic renal disease, bacteraemia of unknown focus and cognitive impairment at admission (OR, 2.812 [95% CI, 1.039-7.611; p = 0.042]; OR, 6.179 [95% CI, 1.840-20.748; p = 0.003]; OR, 8.673 [95% CI, 1.557-48.311; p = 0.014] and OR, 3.621 [95% CI, 1.226-10.695; p = 0.020], respectively). By multivariate analysis appropriate antibiotic therapy was not associated with lower odds of mortality.

**Conclusion:**

Bacteraemia in the elderly has a high mortality rate. There are no set of signs or clinical features that can predict bacteraemia in the elderly. However, older age (≥ 85 years), chronic renal disease, bacteraemia of unknown focus and severe cognitive impairment adversely affects the outcome of elderly patients with bacteraemia admitted to an Internal Medicine ward.

## Background

In Portugal, the proportion of individuals above the age of 65 represents 16% of the population today, but they consume more than 36% of the health care resources [1,2]. As a result many of the patients admitted to an Internal Medicine ward are elderly. These patients are also more susceptible to infections and unusual disease presentations, because of their comorbidities, physical and cognitive debility and a waning immunity [3,4].

Despite the evidence that advances in antibiotic therapy, during the last decades have decreased mortality, the prognosis of elderly patients with infections, especially multi-resistant bacteria, continues to be reserved [5-7]. As a consequence, the knowledge of risk factors that could predict a worse outcome in the infected elderly patients could be used as a support to the clinical decision making. Recent studies have developed scoring systems devised to identify important prognostic factors that influence the patient's outcome after admission [8-13]. Most studies were clinic based and restricted to selected patients groups such as critically ill patients [14] and patients with *Sthaphylococcus aureus *[15] or Enterococcal bacteremia [16].

As few studies [11,15,17,18] have established a risk stratification model to predict outcome in elderly patients with bacteraemia, we retrospectively analysed data from a cohort of selected elderly patients with a confirmed positive blood culture, with the purpose to assess the association between older age, appropriate empirical antibiotic therapy, clinical and laboratory features at admission and in-hospital mortality.

Our hypothesis was that patients would have higher mortality rate the older they became. Inadequate empirical antibiotic therapy and comorbidities would also be independent predictors of mortality in elderly patients with bacteraemia.

## Methods

### Study Design

The University Hospital of Coimbra (UHC) is a Tertiary Hospital that has one emergency department, which was attended by approximately a total of 165.000 people in the year 2009. We conducted a retrospective observational study in a cohort of elderly admitted to one of the four Internal Medicine Wards at the UHC (33 beds), through the emergency department, within 12-month period (1^st ^January till 31^st ^December 2009).

In this study all potential subjects were screened by reviewing the hospital medical record.

### Eligibility Criteria

Eligible subjects were hospitalized in the study Internal Medicine Ward where access to in-depth information about the clinical management and outcome was available.

As inclusion criteria we considered patients with 65 years of age or older admitted to the study Internal Medicine ward because of a suspected infection and with a positive blood culture collected at the emergency department before admission. It was only included in this study bacteraemic episodes acquired outside the hospital settings, with the aim of improving our knowledge in this type of infections.

As exclusion criteria we considered patients with positive blood culture for coagulase negative *Staphylococcus*, *Staphylococcus Epidermidis *and Bacillus species. Patients with HIV infection, active malignancy or taking immunosuppressive drugs, cytotoxic drugs, steroids at a dose ≥ 20 mg/d for more than 2 months) were also excluded, since the risk of infection was already high in these groups.

A total of 381 positive blood cultures results (240 patients) were available from microbiology laboratory in patients aged ≥ 65 years of age. One hundred and eighty (47.2%) positive blood cultures (77 patients) were removed from analysis because the blood culture isolate was determined to be a contaminant. From the remaining 201 positive blood cultures (163 patients) twenty-eight were excluded because patients were taking immunosuppressive drugs or were with active malignancy. Only 175 positive blood cultures from 135 patients were analysed.

### Data Collection

The medical chart of each patient with documented bacteraemia was reviewed by an internal medicine and infectious disease physician and the data recorded was stored in a Microsoft Excel form. This included: demographic data regarding gender, age, length of hospital stay, number of hospital admissions in the previous 90 days, residence prior to admission (i.e. home or long-term care facility), reasons for admission, comorbid illness, source and type of infection. Laboratory data such as: creatinine, glucose, albumin, C reactive protein, haemoglobin, mean globular volume, Leukocytes, arterial blood gas analysis (pH, pO_2_, pCO_2_, HCO_3 _and lactates) and clinical data at admission: blood pressure, temperature (measured in Celsius scale), heart rate, activity of daily living (ADL) score and level of cognitive impairment, were also recorded.

The empirical antibiotic therapy was administered after blood culture collection. It was regarded as appropriate if the blood isolate was susceptible to one or more of the antibiotic drugs given and as inadequate if isolates were found to be resistant (based on the antibiotic susceptibility test results).

### Microbiologic Evaluation

Blood culture specimen was collected within the first 3 hours on admission (in the emergency department) and prior to initiating empirical antibiotic therapy.

Isolates obtained from a standard bacterial, fungal or mycobacterial blood culture automated detection system (Bact/ALERT, 3D microbial detection, bioMérieux, Marcy l'Etoile, France) were identified by the clinical microbiology laboratory database from the UHC.

### Study Definitions

A case of bacteraemia, fungemia or mycobacteraemia was defined as a bacteria or fungus growth in blood culture, in which the isolated pathogen was determined to have an etiological role based on clinical and microbiological assessment.

A modified version of Katz model of functional status assessment [19,20] was used to determine the ADL score. This modified version of the Katz Index is an ordinal index that easily allows a brief assessment of the physical functioning of the elderly and chronically ill patients admitted in the Internal Medicine wards of the UHC. A dichotomous rating (dependent/independent) of six major areas of ADL functions (in order of increasing dependency): bathing, dressing, going to the toilet, transferring from bed to chair, continence, and feeding, assigned patients to a six-point scale of dependence [20]. Cognitive impairment was determined at admission and was based on a ten question questionnaire, the short portable mental status questionnaire (SPMSQ), in which patients were given a score of 1 for each incorrectly answered question [21]. A score 0-2 errors corresponded to a normal cognitive function; 3 to 4 errors was considered mild cognitive impairment, 5 to 8 errors represented a moderate cognitive impairment and more than 8 errors was considered severe cognitive impairment.

The medical comorbidities of interest were classified as follows: cardiovascular disease was considered if a treatment was being provided for coronary artery disease, congestive heart failure, arrhythmias or valvular heart disease; hypertension; pulmonary disease: treatment for chronic obstructive lung disease or interstitial lung disease; chronic renal disease: pre-existing renal disease with documented abnormal creatinine level prior to hospitalization; liver disease: pre-existent viral hepatitis or liver cirrhosis; diabetes mellitus; cerebrovascular disease: acute or chronic vascular and nonvascular encephalopathy and dyslipidemia was defined by LDL ≥ 130 mg/dl, HDL < 40 mg/dl, total cholesterol ≥ 200 mg/dl, or triglycerides ≥ 150 mg/dl.

Sepsis was defined as having at least two of the following four criteria for systemic inflammatory response syndrome (SIRS): body temperature < 36°C or > 38°C, heart rate > 90 bpm, respiratory rate > 30 bpm or pCO_2 _< 32 mmHg, white blood cells < 4.000 cells/mm^3 ^or > 12.000 cells/mm^3 ^and the presence of a known or suspected source of infection [22].

The suspected source of infection was assessed based on the review of the available medical records.

### Primary outcome

The primary outcome of the study was all-cause in-hospital mortality in elderly patients with bacteraemia acquired outside hospital settings.

### Statistical analysis

Categorical data was characterized based on the absolute and relative frequencies. The numeric variables were presented according to their central tendency measures (mean and median) and dispersion (standard deviation and percentiles).

For comparison of the three age group non-parametric test, Kruskal-Wallis and Chi-square tests for trend were used. Survival and death groups were compared by Chi-square test, Fisher's exact test (in the case of small numbers) or Mann-Whitney Test. Receiving operator characteristics (ROC) curve were used to define the cut-off point in a continuous variable. The level of significance for all statistical tests was 2-sided, with α < 0.05.

A stepwise multiple logistical regression model was used to identify the significant associations between the potential predictors and in-hospital mortality. Selection was made among the potential predictors (clinically important and with p value < 0.25 in the univariate analysis) of in-hospital mortality using a stepwise selection method with likelihood ratio test. Significance needed for removal was set at P > 0.10 and significance for reentry at P < 0.05. The size of the effect of these variables was quantified in odds ratio (OR) with 95% confidence interval (CI). SPSS software, version 13.0 (SPSS), was used for analysis.

## Results

A total of 381 positive blood cultures from patients aged ≥ 65 years, admitted to the study ward, were available for review. However, only 179 (47.0%) positive blood cultures (corresponding to 135 bacteraemic episodes) were included in the study.

Thirty-one (23.0%) episodes occurred in patients between the ages 65 and 74; 63 (46.7%) between ages 75 and 84, while 41(30.4%) were in patients ≥ 85 years. More than half (54.1%) were men, however there was trend to an increase in the proportion of women at older ages (29.0% vs 47.6% vs 56.1%; p = 0.026). Ninety-five patients (70.4%) came from home and forty (29.6%) were admitted from long-term care facilities. There were more patients in the age group (≥ 85 yrs) coming from long-term care facilities (16.1% vs 25.4% vs 46.3%; p = 0.004). The median length of hospital stay was twelve days (Table 1).

**Table 1:** Demographics of elderly patients with bacteraemia admitted to an Internal Medicine Ward.

		Age groups							
					
	65-74 yrs (n = 31)		75-84 yrs (n = 63)		≥ 85 yrs (n = 41)		Total		*P*
**Gender: **female n (%)	9	(29.0)	30	(47.6)	23	(56.1)	62	(45.9)	**0.026**
**Admitted from: **n (%)									
Long-term care facility	5	(16.1)	16	(25.4)	19	(46.3)	40	(29.6)	**0.004**
Home	26	(83.9)	47	(74.6)	22	(53.7)	95	(70.4)	
**Length hosp**.: median^a^	15	(9-33)	13	(6-22)	11	(7-26)	12	(7-23)	0.513
**H. admission**^b^: n (%)	17	(56.7)	28	(47.5)	20	(50.0)	65	(48.1)	0.625
**Bedridden state: **n (%)	14	(45.2)	34	(54.0)	33	(80.5)	81	(60.0)	**0.002**
**Comorbidity: **n (%)									
Cardiovascular disease	3	(9.7)	16	(25.4)	7	(17.1)	26	(19.3)	0.535
Pulmonary disease	4	(13.9)	8	(12.7)	6	(14.6)	18	(13.3)	0.817
Liver disease	2	(6.5)	1	(1.6)	1	(2.4)	4	(3.0)	0.367
Chronic renal disease	6	(19.4)	10	(15.9)	5	(12.2)	21	(15.6)	0.405
Cerebrovascular disease	11	(35.5)	36	(57.1)	14	(34.1)	61	(45.2)	0.719
Hypertension	9	(29.0)	21	(33.3)	12	(29.3)	42	(31.1)	0.977
Diabetes Mellitus	10	(32.3)	19	(30.2)	12	(29.3)	41	(30.4)	0.790
Dyslipidemia	0	(0.0)	3	(4.8)	5	(12.2)	8	(5.9)	**0.028**
**Source of Infection: **n (%)									
UTI	12	(38.7)	30	(47.6)	21	(51.2)	63	(46.7)	0.562
LRI	14	(45.2)	23	(36.5)	13	(31.7)	50	(37.0)	0.500
Skin ulcer infection	4	(12.9)	8	(12.7)	6	(14.6)	18	(13.3)	0.817
Bacteraemia^c^	3	(9.7)	2	(3.2)	2	(4.9)	7	(5.2)	0.415
Miscellaneous infections^d^	3	(9.7)	2	(3.2)	4	(9.8)	9	(6.7)	0.357

^a ^Length of hospital stay: median (p25-p75)

^b ^Hospital admission in the previous 90 days

^c ^Bacteraemia of unknown source of infection

^d ^Including gastrointestinal, central nervous system, muscle and bone infection

LRI- Lower respiratory infection

UTI- Urinary tract infection

**Figure 1:**
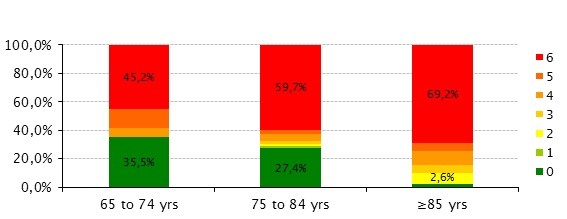
**Evaluation of the Functional status using the modified version of Katz's index of independence on ADL**. Elderly patients (≥ 85 yrs) present more dependence in the ADL (Kruskal-Wallis test, p = 0,051).

**Figure 2:**
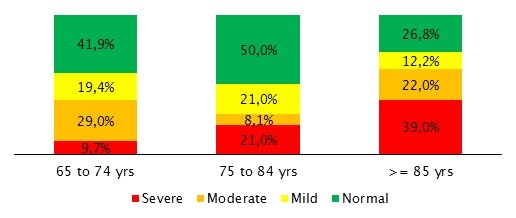
**Evaluation of Cognitive impairment (measured by the SPMSQ scale) among the 3 age groups**.

There was a significantly larger proportion of patients in the bedridden state at the older age group (45.2% vs 54.0% vs 80.5%, p = 0.002). The same was observed with the functional and cognitive status, older patients were more dependent on the ADL (p = 0,051; Figure 1) and presented more cognitive impairment (p = 0.013; Figure 2).

One or more comorbidities were present in 113 patients (83.7%). The most common were: cerebrovascular disease (45.2%), hypertension (31.1%), diabetes mellitus (30.4%), cardiovascular disease (19.3%), chronic renal disease (15.6%) and pulmonary disease (13.3%). A higher proportion of patients at the older age group had dyslipidemia (0.0% vs 4.8% vs 12.2%; p = 0.028).

In our cohort the most common infections were urinary tract infection (UTI) (46.7%) followed by the lower respiratory tract infections (LRI) (37.0%). The source of infection was not identified in 7 patients (5.2%).

A total of 179 microorganisms in the 135 bacteraemic episodes (135 patients) were isolated in the blood cultures. More than one bacterial species were identified in 42 episodes of bacteraemia. Gram positive organisms were more frequently isolated in the blood cultures (54.3%). Seventy-five (42.8%) bacteraemic episodes were caused by aerobic Gram negative bacilli (Table 2). *Escherichia coli (14.9%) *was the microorganism preferentially isolated from blood cultures, followed by Methicillin-resistant *Staphylococcus aureus *(MRSA) (12.0%), Non-MRSA (11.4%), *Klebsiella pneumoniae *(9.1%), *Enterococcus faecalis *(8.0%) and *Pseudomonas aeruginosa *(4.6%). There was a higher number of MRSA isolates in the older age group (3 vs 6 vs 12, p = 0.015). Of the 21 bacteraemic episodes associated with MRSA infections, 9 (6.7%) patients had UTI, 6 (4.4%) had LRI and 4 (3.0%) had MRSA bacteraemia of unknown source of infection.

**Table 2:** Blood culture isolates' distribution by age-groups

	Age groups				
			
	65-74 yrs	75- 84 yrs	≥ 85 yrs	Total*	
	n = 39	n = 81	n = 55	n	(%)
**Gram-positive bacteria**				95	-54.3
*Staphylococcus aureus*:					
- MRSA	3	6	12	21	-12
- NMRSA	8	7	5	20	-11.4
*Enterococcus faecalis*	3	7	4	14	-8
S. *haemolyticus*	0	5	0	5	-2.9
S. *pneumoniae*	0	5	1	6	-3.4
Other Gram positives	8	14	7	29	-16.6
					
**Gram-negative bacteria**				80	-45.7
E. *coli*	5	16	5	26	-14.9
K. *pneumoniae*	2	8	6	16	-9.1
*Pseudomonas aeruginosa*	3	1	4	8	-4.6
Other Gram negatives	6	10	9	25	-14.3
					
**Fungus**	1	2	2	5	-2.9

*Total number of isolates = 175.

### Outcome

Overall there were 30 (22.2%) deaths among the 135 patients. Eight (25.8%) of the 31 patients in the 65-74 age range, nine (14.3%) of the 63 patients in the 75-84 age range and thirteen (31.7%) patients of the 85 and older age group died during hospitalization.

Mortality was examined with regard to the source of bacteraemia. It was greater with bacteraemia of unknown source of infection (57.1%), followed by skin ulcer infection (23.5%), UTI (21.3%) and LRI (14.6%).

Mortality rate for patients with MRSA infection was higher than for those without MRSA infection, but with no statistical difference ((8/21 vs 21/108, p = 0.085).

Of the 135 patients, 93 met the definition of sepsis. Mortality rate between patients with sepsis syndrome and without (21/93 vs 4/21; p = 1.000) was similar.

Further analysis of the partial pressure of oxygen in arterial blood (pO_2_), serum albumin concentration and body temperatures between survivors and non-survivors revealed statistically differences in the mortality rates. The cut-off point for serum albumin taken from the ROC curve was 3.05 g/dl. Levels below 3.05 g/dl (hypoalbuminaemia) showed higher mortality rates when compared with patients with levels above (33.3% vs. 16.0%, p = 0.031). The best cut-off for pO_2 _was 59.65 mmHg. Patients with oxygen levels below 59.65 mmHg (hypoxemia) presented higher mortality rates when compared with those with levels above (30.6% vs. 8.5%, p = 0.01).

In patients with hypothermia (< 36°C), which was rare in the present study (6.7%), there was a 55.6% mortality rate. This compares to patients with temperatures between 36°C to 37.4°C and temperatures 37.5°C or higher, whose mortality rates were 25.9% and 14.5%, respectively (p = 0.016).

### Risk factors for in-hospital mortality

By univariate analysis, the factors associated with increased odds of in-hospital mortality were chronic renal disease, bacteraemia of unknown source of infection, severe cognitive impairment, hypoxemia, hipoalbuminaemia and hypothermia (Table 3). Appropriate empirical antibiotic was associated with a decreased odds of mortality.

**Table 3:** Risk factors associated with increased odds of in-hospital mortality in elderly patients with bacteraemia (univariate analysis).

	Survived n = 105		Died n = 30				
	n	(%)^c^	n	(%)^d^	OR	(95%CI)	*P*
**Gender:**							
Male	55	(52.4)	18	(60.0)	1.0 (reference group)		
Female	50	(47.6)	12	(40.0)	0.733	(0.321-1.673)	0.460
**Age:**							
65-74 yrs	23	(21.9)	8	(26.7)	0.755	(0.266-2.148)	0.097
75-84 yrs	54	(51.4)	9	(30.0)	0.326	(0.120-0.883)	
≥ 85 yrs	28	(26.7)	13	(43.3)	1.0 (reference group)		
**Admission from:**							
Home	74	(70.5)	21	(70.0)	0.977	(0.403-2.372)	0.960
Nursing-home	31	(29.5)	9	(30.0)	1.0 (reference group)		
Bedridden state	57	(54.3)	20	(66.7)	1.676	(0.695-4.045)	0.247
Length of hospital stay	13	(7-22)	12	(40.0)	NA	NA	0.760
Previous Hospital admissions	46	(43.8)	19	(63.3)	2.230	(0.943-5.275)	0.064
Appropiate antibiotic therapy	40	(38.1)	6	(20.0)	0.375	(0.139-1.010)	**0.047**
**Comorbidities:**							
Cardiovascular disease	17	(16.2)	7	(23.3)	1.553	(0.573-4.214)	0.571
Pulmonary disease	15	(14.3)	5	(16.7)	1.181	(0.389-3.578)	0.774
Chronic renal disease	11	(10.5)	8	(26.7)	3.082	(1.103-8.611)	**0.037**
Cerebrovascular disease	48	(45.7)	10	(33.3)	0.570	(0.056-4.023)	0.198
Hypertension	29	(27.6)	10	(33.3)	0.765	(0.331-1.765)	0.571
Diabetes Mellitus	32	(30.5)	8	(26.7)	0.810	(0.324-2.024)	0.651
Dyslipidemia	7	(6.7)	1	(3.3)	0.474	(0.056-4.023)	0.862
**Source of Infection**							
UTI	48	(45.7)	13	(43.3)	0.908	(0.401-2.057)	0.817
LRI	41	(39.0)	7	(23.3)	0.475	(0.187-1.207)	0.113
Skin ulcer infection	13	(12.4)	4	(13.3)	0.462	(0.127-1.679)	1.000
Bacteraemia^a^	3	(2.9)	4	(13.3)	1.071	(0.321-3.575)	**0.045**
Miscellaneous^e^	7	(6.7)	2	(6.7)	1.000	(0.197-5.087)	1.000
**Microorganism**							
MRSA	13	(12.4)	8	(26.7)	2.549	(0.937-6.940)	0.085
NMRSA	15	(14.3)	5	(16.7)	1.181	(0.389-3.578)	0.774
*Enterococcus faecalia*	12	(11.4)	2	(6.7)	0.543	(0.114-2.579)	0.735
*E. coli*	23	(21.9)	3	(10.0)	0.386	(0.107-1.393)	0.135
K. pneumoniae	12	(11.4)	4	(13.3)	1.173	(0.348-3.957)	0.796
P. aeruginosa	4	(3.8)	4	(13.3)	3.840	(0.897-16.438)	0.075
**Physiologic presentation**							
Sepsis (SIRS + infection)	72	(68.6)	21	(70.0)	1.240	(0.376-4.086)	1.000
Functional status (ADL score ≥ 1)	76	(72.4)	26	(86.7)	2.737	(0.761-9.845)	0.111
Cognitive impairment^b^	49	(46.7)	22	(73.3)	3.207	(1.256-8.188)	**0.012**
pO2 ≤ 59.65 mmHg	25	(23.8)	11	(36.7)	4.730	(1.361-16.444)	**0.010**
Albumin ≤ 3.05 g/dl	26	(24.8)	13	(43.3)	2.615	(1.072-6.380)	**0.031**
Temperature:							
< 36°C	4	(3.8)	5	(16.7)	1.0 (reference group)		
36-37.5°C	43	(41.0)	15	(50.0)	0.279	(0.066-1.178)	**0.016**
> 37.5^a^C	53	(50.5)	9	(30.0)	0.136	(0.031-0.604)	

^a ^Bacteraemia without an identifiable source of infection

^b ^moderate to severe cognitive impairment defined as having a SPMSQ score > 4

^c, d ^percentage calculated on the basis of the total number of survivors and deaths, respectively

^e ^Including central nervous system, muscle and bone infection

CI = Confident interval, LRI- Lower respiratory infection, UTI- Urinary tract infection, SIRS- systemic inflammatory response syndrome, NA- not available

**Figure 3:**
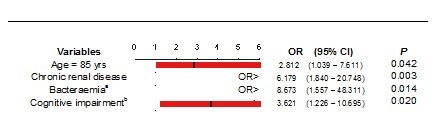
**Risk factors associated with in-hospital mortality in elderly patients with bacteraemia (Multivariate logistic regression analysis)**. ^a ^Bacteraemia without an identifiable source of infection. ^b ^moderate to severe cognitive impairment defined as having a SPMSQ score > 4.

A multivariate analysis was also conducted, age ≥ 85 years was identified as independent predictor of mortality (OR, 2.812 [95% CI, 1.039-7.611], p = 0.042). Chronic renal failure, bacteraemia of unknown source of infection and severe altered mental status were also identified as risk factors for mortality (OR, 6.179 [95% CI, 1.840-20.748], p = 0.003], OR, 8.673 [1.557-48.311], p = 0.014] and OR, 3.621 [1.226-10.695], p = 0.020], respectively) (Figure 3).

## Discussion

In the last decades several studies have reported changes in microbiology, epidemiology and outcomes in adults with bacteraemia [23-25]. Some of these studies have already reported variable clinical presentation of infectious diseases in the elderly [26,27]. Traditional markers of infection severity are frequently blunted or absent [28]. Fever is uncommon, while cognitive impairment is frequent and therefore, high index of suspicion is warrant in these patients.

The main purpose of our study was to identify independent predictors of in-hospital mortality in elderly patients with bacteraemia acquired outside the hospital settings.

A high percentage of the positive blood cultures were excluded from the study because isolates were considered contaminant (180/381 = 47.2%). Most of these contaminants were coagulase-negative *staphylococcus *bacteria. These high percentages have already been seen in previous studies [29,30]. These underline the need for careful collection of the blood specimens at the ED to avoid frequent contaminants.

Consistent with previous reports, the urinary tract infections were the most common source of bacteraemia [18,31,32].

As in other studies, E. *coli *(14.9%) and MRSA (12.0%) remain the most frequently isolated bloodstream pathogens [11,23,31]. Anaerobic bacteraemia and fungemia were uncommon. In contrast to previous reported series of bacteraemia, the majority of isolates, in our study, were Gram positives (54.3%) [2,8,30]. This increase in prevalence is probably due to the institutionalization of many elderly patients, and an increased early use of broad spectrum antibiotics, which selects for more virulent and resistant strains such as MRSA. Therefore, the relative frequency of MRSA has been increasing, whereas bacteraemia due to S. *pneumonia *has been decreasing at the Internal Medicine wards. S. *pneumoniae *was only detected in 3.4% of the total isolates. This high prevalence of MRSA highlights the importance of wide empiric antibiotic coverage if MRSA bacteraemia is suspected, whether the type of infection is acquired inside or outside the hospital settings.

As in previous studies [33-35], in our study older age was associated with significantly increased rates of mortality (OR, 2.812 [95% CI, 1.039-7.611], p = 0.042). Older patients are often nutritionally or immunologically impaired, making them an easy target for infection and its associated complications [36]. Moreover, the increased mortality observed in elderly patients with bacteraemia may also be due to the increased comorbidities among this age group.

Various physiologic parameters estimated at the time of presentation were analysed to find predictors of mortality. Only moderate to severe cognitive impairment (SPMSQ score > 4) at admission of the Internal Medicine ward was found to be independent predictor of mortality. Severe cognitive impairment may lead patients to a bedridden state and consequently to the development of decubitus skin ulcers and bloodstream infections.

A high proportion of patients with one or more dependence on ADL were observed in the older age group. The consequences of severe physical inability and cognitive impairment include the inability to be able to self-care and depression of consciousness, which may result in nutritional or immunologic deficits or both and impair resistance to infection [37]. Functional status has been recognized has a predictors of mortality in some studies [37-39], but this association was not demonstrated in our study.

In the present study, although hypoxemia and hypoalbuminaemia were not identified as independent predictors of mortality by multivariate analysis, patients who died tended to present lower levels of albumin and pO_2_. This difference was only statistically significant in univariate analysis. Hypoalbuminaemia had already been reported by Greenberg et al. as associated with increased rates of mortality in elderly patients with bacteremia [11]. Many possible explanations for this finding have been formulated including albumin as a general indicator of health status and immunologic competence. Low albumin levels may also indicate that the patient has lower physiologic reserve for handling stresses such as infections and bacteraemias.

Hypoxemia has also been identified, in a recent prospective cohort study, as an independent risk factor for death [27]. In our study hypoxemia was significantly associated with death in univariate analysis (OR, 4.730 [95% CI, 1.361-16.444], p = 0.010), but not in multivariate analysis.

Hypothermia was associated with significantly increased rates of mortality (p = 0.016). The reason for this observation is still not known, but many authors speculate that may be related to the fact that the lack of fever may delay blood cultures collection and antibiotic therapy intervention [23,24]. Other studies have demonstrated that neutrophil migration, T-cell proliferation and production of interferon and other cytocines are enhanced in the presence of fever [31]. Therefore, lack of fever may compromise the immunological response to bacteraemia.

In the multivariate analysis we also detected increased odds of mortality with chronic renal disease. This finding has also been observed in other studies [9,11].

In the present study appropriate antibiotic therapy was associated with decreased odds of mortality in univariated analysis but not in multivariated analysis, as it had already been reported in other series [11,40,41]. Some systematic reviews have already assessed the impact of appropriate antibiotic therapy in mortality rates in bacteremic patients [42-44]. They have shown that some of the limitations regarding this issue are related to the fact that variables that influence the efficacy of antibiotics *in vivo *are not considered in the multivariate analysis. The variables related to the pharmacokinetics of the antibiotics, clinical severity of the bacteraemia and follow-up duration are not always measured or adjusted for the final analysis. This can lead to a biased estimate of the real effect. McGregor *et al. *identified severity of illness, patient comorbidity and the presence of polymicrobial bacteremia as potential confounders of the association between appropriate antibiotic therapy and mortality [42].

This study has some limitations regarding the fact of being a retrospective analysis based upon a chart review, which may be incomplete in the ED setting. The study was conducted in only one hospital, and practice patterns, patients' characteristics microbiology resistance pattern may vary among hospitals. There was some data not available for all patients and it is possible that some bias may have been introduced. Information on nutrition status was not available, but should ideally be included, since this may influence the risk of infection [8].

Although the use of multivariate analysis helped to control a substantial proportion of any confounding variable, data related to the severity of the illness and prior use of antimicrobials was not reported and therefore not included in multivariated analysis, which may have underestimated the efficacy of appropriate antibiotic treatment on mortality rates.

This study used a methodology similar to other studies already published and performed in elderly patients [11,31,45]. It provided insight into current microbiology, epidemiology and outcomes of elderly bloodstream infections

## Conclusion

Elderly patients are more likely to have comorbidities, problems associated with institutionalization or having invasive devices, making them more prone to infections. The current study revealed that in elderly patients with bacteraemia, the most frequent signs and symptoms of infection appear not to have high specificity. Therefore, predicting mortality is particular challenging in these patients.

This study identified four independent risks factors for all-cause in-hospital mortality: age over 85 years, chronic renal disease, severe cognitive impairment and fever of unknown source of infection.

The prognostic factors identified may help in the early recognition of elderly with increased risk of adverse outcome, in the ED.

## Abbreviations

OR: Odds ratio; CI: confidence interval; ED: emergency department.

## Competing interests

The authors declare that they have no competing interests.

## Authors' contributions

MR conceived the study, participated in the design of the study, acquisition and interpretation of data and performed the statistical analysis. BP participated in the design of the study, acquisition and interpretation of data. JL and JM participated in the gathering of data. JV participated in the study design, coordinated and revised the manuscript for important intellectual content. JNC revised the manuscript for important intellectual content.

All authors read and approved the final manuscript.
